# Pharmacotherapy against Oxidative Stress in Chronic Kidney Disease: Promising Small Molecule Natural Products Targeting Nrf2-HO-1 Signaling

**DOI:** 10.3390/antiox10020258

**Published:** 2021-02-07

**Authors:** Md Jamal Uddin, Ee Hyun Kim, Md. Abdul Hannan, Hunjoo Ha

**Affiliations:** 1 Graduate School of Pharmaceutical Sciences, College of Pharmacy, Ewha Womans University, Seoul 03760, Korea; hasan800920@gmail.com (M.J.U.); pionhyun@gmail.com (E.H.K.); 2 ABEx Bio-Research Center, East Azampur, Dhaka 1230, Bangladesh; hannanbmb@bau.edu.bd; 3 Department of Biochemistry and Molecular Biology, Bangladesh Agricultural University, Mymensingh 2202, Bangladesh

**Keywords:** chronic kidney diseases, oxidative stress, Nrf2, HO-1, small molecule natural products

## Abstract

The global burden of chronic kidney disease (CKD) intertwined with cardiovascular disease has become a major health problem. Oxidative stress (OS) plays an important role in the pathophysiology of CKD. The nuclear factor erythroid 2-related factor 2 (Nrf2)-antioxidant responsive element (ARE) antioxidant system plays a critical role in kidney protection by regulating antioxidants during OS. Heme oxygenase-1 (HO-1), one of the targets of Nrf2-ARE, plays an important role in regulating OS and is protective in a variety of human and animal models of kidney disease. Thus, activation of Nrf2-HO-1 signaling may offer a potential approach to the design of novel therapeutic agents for kidney diseases. In this review, we have discussed the association between OS and the pathogenesis of CKD. We propose Nrf2-HO-1 signaling-mediated cell survival systems be explored as pharmacological targets for the treatment of CKD and have reviewed the literature on the beneficial effects of small molecule natural products that may provide protection against CKD.

## 1. Introduction

The incidence and prevalence of chronic kidney disease (CKD) patients is increasing worldwide. The prevalence of CKD between male and female patients is not constant between countries, however, kidney functions decline faster in males than females [1]. Importantly, CKD is not only a risk factor for increasing global mortality but it is also a critical factor involved in cardiovascular disease (CVD) [2]. The close link between CKD and CVD has been known for a long time [3,4,5]. Not only traditional risk factors such as hypertension, dyslipidemia, and diabetes, but also non-traditional risk factors such as disturbed minerals and vitamins in CKD may play important roles in the progression of CVD. The current treatment options for CKD are controlling blood pressure, serum glucose, and serum lipid profile [6], as well as a modification of lifestyle [7,8]. Since the efficacy of the current therapeutic strategy is still limited [9], there is a need to develop a more effective therapeutic option for treating CKD. Although the exact mechanism involved in the development of CKD is elusive, many lines of evidence strongly suggest that oxidative stress (OS) plays a critical role in the progression of CKD [10,11,12,13].

OS is an imbalance between cellular reactive oxygen species (ROS) levels and antioxidant enzymes, leading to a pathological condition. ROS regulates various signaling pathways, including the growth and differentiation of cells, mitogenesis, production, and breakdown of the extracellular matrix (ECM), inflammation, and apoptosis [14]. OS-mediated damaging effects of cells are controlled by activating the antioxidant defense system. OS has also been noticed to be affected by sex hormones in ischemic kidney injury [15]. Unfortunately, there is an impairment of antioxidative defense and a reduced activity of antioxidant enzymes in CKD [16]. Hence, promoting the endogenous antioxidants defense system may become an important strategy in inhibiting OS-mediated cellular damage in CKD.

Phytochemicals and other natural products are cytoprotective against OS by scavenging oxygen-free radicals and enhancing the level of antioxidants [17]. The literature on protective effects of antioxidant natural products against CKD has been reported [18,19,20]. Nuclear factor erythroid 2-related factor 2 (Nrf2) is the master regulator of the cellular antioxidant defense system [17]. Studies review that augmentation of Nrf2 activity prevents the progression of acute kidney injury (AKI) to CKD transition [21,22]. Natural bioactive compounds and their sources have been demonstrated to have kidney protective potential by activating Nrf2 in experimental CKD models [23,24]. In a recent review on clinical studies, bardoxolone methyl (CDDO-me), a semi-synthetic triterpenoid activating the Nrf2 pathway, has been reported as an effective therapeutic for diabetic kidney disease (DKD), although it has limitations in that it increases the risk of heart failure [25]. Heme oxygenase-1 (HO-1), one of the target molecules of Nrf2, attenuates the overall production of ROS through its ability to degrade heme and to produce carbon monoxide (CO), biliverdin/bilirubin, and the release of free iron. Induction of HO-1 mediates many beneficial effects in the cardiovascular system and kidney [26]. Also, the modulatory role of HO-1 has been reported in various kidney injury models including CKD [27,28,29,30,31,32,33,34]. Several natural HO-1 inducers and their therapeutic applications in various diseases, including CKD, have been reported [35].

In this review, we have explored the causes responsible for the development of OS and its involvement in the pathophysiology of CKD. We then introduced Nrf2-HO-1 signaling as pharmacological targets for the treatment of CKD. Finally, we have discussed the recent literature on its protective effects on the kidney and the underlying pharmacological mechanisms of bioactive phytochemicals that activate Nrf2-HO-1-mediated kidney protective actions.

## 2. Oxidative Stress in Chronic Kidney Disease

**Figure 1:**
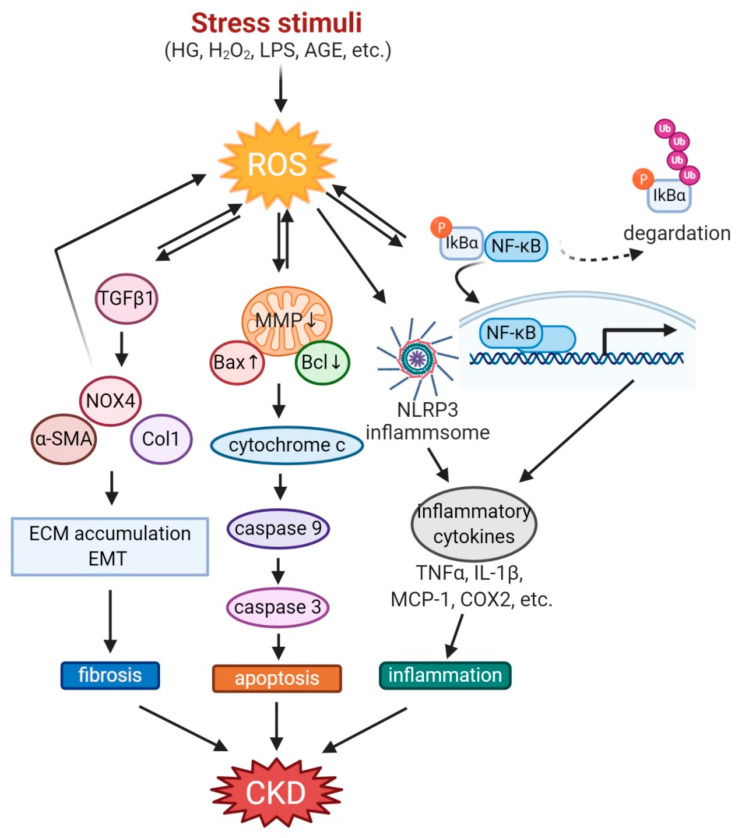
Mechanisms involved in the pathogenesis of OS in CKD. Stress stimuli such as high glucose, H_2_O_2_, and AGE generate ROS in the kidney. The higher ROS increases TGFβ activation, causing kidney fibrosis through the accumulation of ECM and EMT. ROS also decreases MMP and regulates Bax/Bcl2 in mitochondria to activate caspase3, resulting in apoptosis. Not only nuclear localization of NF-kB through degradation of phosphorylated IkBα but also NLRP3 inflammasome activation induces inflammation with inflammatory cytokines secretion. These ROS-induced pathophysiologic conditions exacerbate CKD. AGE, advanced glycation end products; CKD, chronic kidney disease; COX2, cyclooxygenase 2; ECM, extracellular matrix; EMT, epithelial-to-mesenchymal transition; HG, high glucose; LPS, lipopolysaccharide; MCP-1, monocyte chemoattractant protein-1; MMP, mitochondrial membrane permeability; NLRP3, NLR family pyrin domain containing 3; ROS, reactive oxygen species; TNFα, tumor necrosis factor α; TGFβ, transforming growth factor β.

OS plays a critical role in the progression of CKD [36,37], including diabetic kidney disease (DKD), glomerulosclerosis, glomerulonephritis, lupus nephritis (LN), systemic lupus erythematosus (SLE), tubulointerstitial fibrosis, and chronic renal allograft dysfunction (CRAD). The mechanistic link between OS and CKD has been widely investigated [10,11,12]. Briefly, mitochondria and NADPH oxidases are the important sources of intracellular ROS which activate signal transduction cascade and transcription factors, leading to upregulation of genes and proteins involved in remodeling of ECM in the diabetic kidney [10]. In addition, ROS disturb the excretory function of the nephron, leading to homeostasis imbalance and accumulation of metabolic products. ROS also disturb regulatory mechanisms of kidney, such as tubular glomerular feedback, myogenic reflex in the arteriole, and the renin–angiotensin–aldosterone system. As a result, the kidney fails to compensate for water–electrolyte and acid–base imbalances, leading to an additional increase in OS. Ultimately, the progression of CKD is occurred with a variety of complications [11]. This section reviews the mechanisms involved in the different models of CKD, as below and in Figure 1.

### 2.1. Diabetic Kidney Disease (DKD)

DKD is a key microvascular complication of diabetes. NADPH oxidases (Nox)-derived ROS play an important role in inflammation and in the accumulation of ECM in DKD. Both hyperglycemia and dyslipidemia play roles in OS and mitochondrial dysfunction [38,39]. In mice, streptozotocin (STZ) increases the ROS level and the expression of monocyte chemoattractant protein-1 (MCP-1), tumor necrosis factor α (TNFα), macrophage marker (F4/80), transforming growth factor β (TGFβ), and fibronectin (FN). Also, STZ treatment induces mitochondrial and peroxisomal dysfunction. These effects are decreased by APX-115, a pan-Nox inhibitor [40], indicating the involvement of OS in mitochondrial and peroxisomal dysfunction. Increased ROS levels and oxidative DNA damage were found to be associated with increased TGFβ1 and ECM accumulation in STZ-induced diabetic mice [41]. In STZ-induced diabetic rats, methylglyoxal increased the levels of ROS, and Bax, cytochrome C, caspase-9, and caspase-3 expression, while it decreased Bcl2 expression in the kidneys [42]. In high-fat diet (HFD)-induced type 2 diabetic mice, accumulation of kidney OS markers such as 8-isoprostane and 4-hydroxynonenal (HNE) were associated with 12/15-lipoxygenase upregulation, resulting in prediabetic nephropathic phenomenon [43]. HFD-induced OS is contributed by inducible nitric oxide synthase (iNOS) and Nox4, as well as mitochondrial oxidants, induced kidney fibrosis, and glomerular hypertrophy in mice [44]. The Zucker diabetic fatty rat, a model of type 2 diabetes, shows increased ROS, nitric oxide, and lipid and protein peroxidation levels in the kidney associated with alterations in glutathione (GSH)-dependent metabolism and mitochondrial function, leading to the development of kidney injury [45]. In db/db type 2 diabetic mice, treatment with an inhibitor of OS, biliverdin, has also been identified as a potential therapeutic option for translation into clinical application [46]. Under high glucose (HG), peripheral blood mononuclear cells showed decreased catalase (CAT), CuZn superoxide dismutase (SOD), and glutathione peroxidase (GPx) mRNA expression in patients with type 1 diabetic nephropathy [47]. Besides, OS as indicated by the plasma carbonyl group [48], nitrotyrosine [49], and malondialdehyde (MDA) [50] were increased in type 2 diabetic patients.

### 2.2. Glomerulosclerosis and Glomerulonephritis

Glomerulosclerosis is a common final pathological feature of CKD. In Charles Dawley rats, doxorubicin-induced glomerulosclerosis (glomerular podocyte damage) increased proteinuria and reduced their body weights. Doxorubicin increased OS, as indicated by neutrophil cytosolic factor 1 (p47phox) and Nox2 (p91phox) mRNA. Increased oxidative enzyme expression was accompanied by increased lipid peroxidation, as demonstrated by HNE and increased protein nitrosylation demonstrated by nitrotyrosine in doxorubicin nephropathy in Charles Dawley rats. Besides, fibrosis markers such as TGFβ1, CTGF, and Col1α1 mRNA were also upregulated [51].

Acute glomerulonephritis (AGN), an inflammatory and proliferative glomerular disease, is characterized by marked proliferation of mesangial and endothelial cells in the glomerulus, together with infiltration of neutrophils. During the disease process, ROS levels might be increased by neutrophils, monocytes, and mesangial cells. Thus, OS may play an important role in the pathogenesis of AGN [52]. The involvement of OS with experimental AGN is further supported by an increase in lipid peroxidation products and alterations in antioxidants [53]. Membranous glomerulonephritis (MGN) is a nephrotic syndrome that may lead to CKD. In cationic bovine serum albumin (BSA)-induced MGN in the rat, kidney dysfunction and histopathological changes were observed, while oxidants such as MDA were enhanced with a decline in the level of antioxidants, including SOD, CAT, and GPx [54]. Altogether, the oxidant–antioxidant imbalance may cause the development of pathogenic alterations in these glomerulonephritis models.

### 2.3. Lupus Nephritis (LN) and Systemic Lupus Erythematosus (SLE)

LN is a severe and frequent complication of SLE. The important role of OS in LN has been previously described [55]. The New Zealand black/white F1 lupus-prone mice showed impaired kidney function with severe kidney lesions and increased ROS-mediated OS. Besides, they showed enhanced expression of the NLRP3 inflammasome as well as NF-kB activation [56]. The pristine-induced lupus nephritic mice resulted in LN with glomerular oxidative damage associated with an increase in ROS, TGFβ1, FN, and iNOS [57]. A recent review has also explained the role of OS in SLE. Here, mitochondrial dysfunction increased OS, leading to lupus pathogenesis [58]. In lupus nephritic patients, immunohistochemistry analysis of the kidney showed an increased accumulation of 8-oxo-dG [57].

### 2.4. Tubulointerstitial Fibrosis

Tubulointerstitial fibrosis is the most important feature associated with progressive CKD. The remnant kidney of 5/6 nephrectomy-induced CKD in Sprague-Dawley rats showed increased profibrotic cytokines, lipid peroxidation, GSH depletion, and Nox, suggesting the involvement of OS with tubulointerstitial fibrosis [48]. The involvement of OS in kidney tubulointerstitial inflammation and fibrosis in a unilateral ureteral obstruction (UUO) mouse model has been established for a long time. DNA oxidant markers such as 8-oxo-dG and lipid peroxidation markers such as MDA are increased in UUO kidneys. Also, the increase in ROS levels and a reduction in antioxidants, including SOD and CAT, were found to be involved with TGFβ-mediated inflammation and fibrosis [59]. Adenine administration significantly increased the plasma and urinary OS biomarkers and caused morphological and histological damage in the kidney tubules in rats. Adenine also increased the inflammatory biomarkers and reduced the levels of antioxidant enzymes [60,61].

### 2.5. Chronic Renal Allograft Dysfunction (CRAD)

CRAD is defined as a progressive kidney dysfunction with features on biopsy of chronic allograft nephropathy. F344 rat kidneys were orthotopically transplanted into Lewis rats. At 12 weeks following surgery, the kidney expressed a higher level of MDA and lower levels of SOD activity, with increased levels of pathological damaging biomarkers such as serum creatinine levels and the infiltration of tubulointerstitial mononuclear cells, indicating the role of OS in CRAD [62].

### 2.6. The Role of Oxidative Stress in Kidney Cells

Various kidney cells are used to dissect the molecular mechanisms involved in CKD. In mouse proximal tubular epithelial (mProx) cells, Ang II induced hypertrophy. Ang II induced superoxide anion (O_2_^●−^) in cultured tubular epithelial cells. This induction of O_2_^●−^ might be due to p22phox-mediated activation of Nox [63]. In human proximal tubular epithelial (HK-2) cells, lipopolysaccharide (LPS) enhanced the expression of Nox4 and iNOS, leading to an increase in the levels of nitric oxide and O_2_^●−^. Subsequently, these ROS reduced cytochrome C oxidase activity and caused mitochondrial dysfunction by interrupting mitochondrial oxidative phosphorylation [64]. HG increased ROS-induced phosphoinositide 3-kinase (PI3K)/ protein kinase B (Akt)/ glycogen synthase kinase 3β (GSK3β) activity and accelerated epithelial-to-mesenchymal transition (EMT) in HK-2 cells [65]. Treatment with 4-hydroxy-2-hexenal increased ROS levels and increased ERK and JNK expression, triggering NF-kB activation and IkBα degradation in HK-2 cells. Eventually, activation of NF-kB promoted apoptosis by inhibiting Bcl2 and increasing Bax expression [66]. H_2_O_2_ increased levels of ROS in the cytosol and mitochondria, leading to apoptosis in HK-2 cells [67]. Treatment with TGFβ1 increased the levels of ROS and decreased the levels of GSH in HK-2 cells. TGFβ1 stimulated the expression of EMT genes, FN, and collagen1 [68]. When Madin-Darby canine kidney epithelial cells were exposed to oxalate, a constituent of many kidney stones, phospholipase A2 was activated and the ROS level was increased with depolarization of the mitochondrial membrane potential, implying mitochondrial dysfunction through OS is involved in oxalate toxicity [69]. In rat kidney epithelial (NRK-52E) cells, methylglyoxal increased the ROS levels, along with inducing increased expression of Bax, cytochrome C, caspase-9, and caspase-3, while it decreased the mitochondrial membrane permeability and Bcl2 expression [42].

HG induces intracellular ROS in mProx cells and mesangial cells. Also, ROS are induced in glomerular mesangial by advanced glycation end products (AGE) and cytokines. This study suggests that Nox may play a role in ROS generation, leading to DKD [10]. In rat kidney mesangial cells, the role of OS was examined via alleviation of AGE-induced activation of NF-kB, protein kinase C (PKC) activity, and TGFβ1 transcription with antioxidants such as vitamin E and nitecapone [70].

HG induces micro-vesicles generation (which may cause the pathogenesis of many diseases, such as CVD and diabetes) through the ROS/Nox4 pathway in mouse podocyte clone 5 cells. Also, HG-induced micro-vesicles were significantly decreased after pretreatment with N-Acetyl-l-cysteine (NAC, an antioxidant) [71]. In mouse podocyte cell lines, exposure to aldosterone elevated ROS levels. Treatment with NAC prevented OS and attenuated podocyte injury by increasing nephrin expression and inhibiting apoptosis [72].

## 3. Role of Nrf2 and HO-1 against Oxidative Stress

Cellular antioxidant defense systems against OS include SOD, CAT, sulfiredoxin, thioredoxin, γ-glutamine cysteine ligase and synthase, NADPH quinone oxidoreductase (Nqo1), Nrf2, and HO-1. The cellular antioxidant defense system is mainly controlled by the Keap1-Nrf2 pathway, which is activated by stimuli such as electrophilic compounds, ROS, and ER stress. The transcription factor Nrf2 is a master regulator of this system. Under normal conditions, the function of Nrf2 is negatively regulated by Keap1 [73], which promotes its degradation via the ubiquitin-proteasome system [74]. Under stress conditions, after a conformational change in Keap1, Nrf2 is released from the proteasome pathway and it translocates into the nucleus [75]. In the nucleus, Nrf2 binds to the gene regulator antioxidant responsive element (ARE) region and mediates the transcription of antioxidant genes [76]. The association of Nrf2 with kidney diseases has been described in various reports. In brief, Nrf2 plays an important role in improving STZ-induced DKD in mice [41]. In this study, Nrf2−/− mice showed increased ROS levels and higher oxidative DNA damage. Besides, increased TGFβ1 and ECM accumulation were found in diabetic mice. These all were decreased by Nrf2 activation [41]. A study reviewed that genetic or pharmacological augmentation of Nrf2 activity reduces OS in the kidney tubules and significantly prevents the progression of AKI to CKD transition [21]. This review explained that a deficiency of Nrf2 accelerates kidney injury in various models, such as LN, and STZ-induced DKD. On the other hand, a deficiency of Keap1 reduced tubular injury in ischemia reperfusion injury (IRI) and diminished kidney fibrosis in UUO [21]. A recent study suggested a role of GSK3β overexpression-mediated Keap1-independent regulation of the Nrf2 antioxidant response against the folic acid-induced AKI to CKD transition in mice [22]. In LN patients, immunohistochemistry analysis of the kidney showed an increased accumulation of 8-oxo-dG, while the expression of Nrf2 and Nqo1 was decreased [57]. The pristine-induced LN mice had glomerular oxidative damage, while the Nrf2-deficient mice had accelerated kidney damage with an increase in ROS, TGFβ1, FN, and iNOS, suggesting a role of Nrf2 in the regulation of ROS levels [57]. In cationic BSA-induced MGN in the rat, kidney dysfunction and histopathological changes were observed. Also, Nrf2 expression and its downstream antioxidants were responsible for the protective response against OS in MGN rats, while negative regulators of Nrf2, such as Keap1 and oxidants such as MDA, were enhanced, with a decline in the level of antioxidants, including SOD, CAT, and GPx [54]. HG increased ROS-induced PI3K/Akt/GSK3β activity and accelerated EMT in HK-2 cells. Further, the accumulation of EMT was reduced by treatment with an Nrf2 activator, sulforaphane, emphasizing the therapeutic potential of targeting Nrf2-HO-1 signaling [65]. The TGFβ1-increased ROS levels, EMT genes, FN, and collagen 1, were further increased by Nrf2 knockdown and suppressed by Keap1 knockdown in HK-2 cells [68], suggesting a critical role of Nrf2 in kidney fibrosis.

HO-1 is one of the targets of Nrf2 and it degrades heme into CO, iron (induction of ferritin), and biliverdin-IXα [77], all of which have antioxidative, anti-inflammatory, and cytoprotective effects against various diseases, including kidney diseases [78,79,80,81,82,83,84]. Cobalt protoporphyrin (CoPP) and curcumin, inducers of HO-1, induce NQO1 expression in HepG2 cells. In addition, endogenous CO, a by-product of HO-1, induces NQO1 expression [85]. Treatment of CoPP increases the SOD expression and catalase activity in STZ-treated rats [86]. These observations suggest a major and functional role of HO-1 in antioxidant defense. HO-1 has been found to be effective against several injurious stimuli and many clinically relevant diseases such as sepsis, hypertension, atherosclerosis, and acute lung and kidney injury [87,88]. Hyperglycemia is the major reason for increased ROS levels, while it is also an important cause of CKD [89]. High levels of glucose inhibit HO-1 activity [90], which leads to increased OS in the vascular system [91]. Decreased HO-1 expression and activity are observed in type 1 diabetes [92], and upregulation of HO-1 reduces diabetic vascular dysfunction [93]. All of these observations suggest that decreased HO-1 expression and activity in the vascular system may lead to the progression of CKD, since vascular abnormalities are strongly linked with kidney dysfunction [3,4].

Induction of HO-1 improves hyperhomocysteinemia-induced AKI to CKD progression in mice. In this study, administration of the HO-1 inducer, cobalt protoporphyrin-IX, significantly hampered ROS and kidney fibrotic lesions [27]. Hemin, another HO-1 inducer, also improved the kidney function and decreased the expression of markers of OS (as indicated by the levels of MDA) in IRI [28]. Ginkgo biloba extract attenuated the production of ROS in HG-stimulated podocytes, and HO-1 inhibitor treatment abolished these effects [29]. There is a strong link between HO-1 and mitochondrial function [30]. Mitochondria-targeted HO-1 attenuates ROS in kidney epithelial cells [31]. HO-1 induction is cytoprotective against ROS-mediated OS in the kidney through an increase in the levels of mitochondrial transporters and cytochrome c oxidase activity [30]. HO-1 overexpression decreased ROS levels, suggesting a decrease in levels of Nox, a heme-dependent protein [32]. Therefore, the induction of HO-1 results in a superior cellular environment due to its good antioxidant capabilities.

## 4. Functional Link between Nrf2 and HO-1

**Figure 2:**
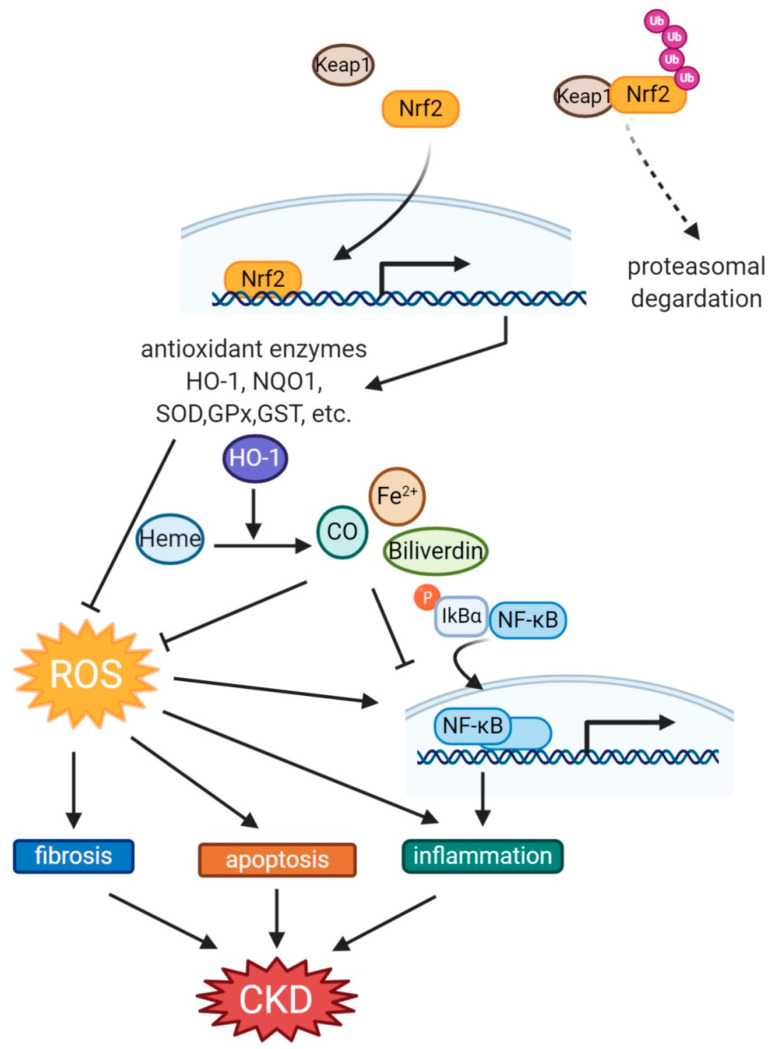
The protective mechanism via the Nrf2-HO-1 pathway on CKD. When Keap1, which targets Nrf2 for ubiquitination and proteasomal degradation, is decreased or inactivated, phosphorylated Nrf2 is translocated into the nucleus, and transcription of downstream antioxidant enzymes is activated. In particular, by HO-1, fibrosis, apoptosis, and inflammation are attenuated either directly or indirectly through inhibition of OS, thus improving kidney damage. CKD, chronic kidney disease; CO, carbon monoxide; GPx, glutathione peroxidase; GST, Glutathione-S-transferase; HO-1, Heme oxygenase-1; Nqo1, NADPH quinone oxidoreductase; Nrf2, nuclear factor erythroid 2-related factor 2; SOD, superoxide dismutase; ROS, reactive oxygen species.

Since under electrophiles and ROS stress Keap1 is modified at its cysteine residues, Nrf2 is released and translocated to the nucleus and binds to the ARE regions [94]. As a result, Nrf2 induces transcription of ARE regulatory genes, including HO-1 in various tissues and cells under OS conditions [95,96,97,98]. Considering the important cytoprotective role of HO-1 in the kidney and other tissues [33,34], in this review, we have focused on the Nrf2-HO-1 pathways (Figure 2).

Nrf2-induced HO-1 may protect the kidney from remote organ injury in mice and rats [99]. HO-1 induction could be a potential therapeutic approach to prevent CKD complications by activating antioxidative and antiapoptotic signaling. Yoh and colleagues speculate that the decrease in HO-1 expression might be associated with the pathogenesis of LN in Nrf2−/− female mice [100]. Also, adenine-induced OS and inflammation were associated with kidney tubulointerstitial fibrosis with impairment of Nrf2 activation and downregulation of its target gene products, including HO-1 [60]. Small molecule activator of Nrf2, CDDO-Im (1-[2-cyano-3-,12-dioxooleana-1,9(11)-dien-28-oyl] imidazole), protects against LPS-induced dysregulation of the innate immune response in mice. Specifically, CDDO-Im treatment reduced LPS-induced ROS levels and inflammatory cytokines such as TNFα and IL-6, as well as increased antioxidative genes such as HO-1, GCLC, GCLM, and Nqo1 in Nrf2+/+ neutrophils but not Nrf2−/− neutrophils in mice [101]. Also, CDDO-Me treatment attenuates retinal vascular degeneration following IRI and increases the expression of HO-1 in wild-type, but not Nrf2−/−, retinas in mice [102], suggesting that activation of Nrf2-dependent compensatory antioxidative pathways by CDDO compounds may protect tissues or cells from OS-induced injury.

The protective effect of insulin-mediated HO-1 was through the PI3K/Akt pathway and the Nrf2 transcription factor in mProx cells [103]. In HK-2 cells, the Nrf2 activator sulforaphane increased HO-1 protein expression. HG increased the expression of vimentin and FN and decreased E-cadherin expression, which were attenuated by the treatment with sulforaphane [65], indicating an inhibitory effect of Nrf2-HO-1 on HG-induced EMT. In mouse mesangial cells, Nrf2 overexpression upregulated Nrf2 and its downstream HO-1 expression under HG, while Nrf2 siRNA-treatment reduced the expression of Nrf2 and HO-1, leading to an increase in ROS and TGFβ1 [61,104]. In another study, Nrf2 deficiency upregulated the NF-kB and TGFβ1 signaling pathway and decreased the expression of downstream antioxidants of HO-1 and Nqo1 in mouse mesangial cells [57], indicating that Nrf2-dependent HO-1 expression limits the activation of NF-kB and inhibits pro-inflammatory cytokines production. Besides, in human umbilical vein endothelial cells (HUVECs), 3-hydroxyanthranilic acid (HA) treatment increases ARE-driven luciferase activity. TNFα-induced NF-kB/DNA-binding activity was suppressed by HA-induced Nrf2 transcription and HO-1 activity, and the NF-kB/DNA-binding activity was restored by treatment with tin protoporphyrin IX dichloride (SnPP, an HO-1 inhibitor) [105]. In HUVECs, constitutively active PKCε enhanced HO-1 mRNA and protein levels, while aortas or cardiac endothelial cells from PKCε-deficient mice showed decreased levels of HO-1. Also, Ang II stimulated PKCε and produced HO-1 in a PKCε-dependent manner. Nrf2 siRNA blocked PKCε-mediated HO-1 induction [106]. In rat pheochromocytoma PC12 cells, dominant-negative Nrf2 significantly inhibited PI3K-induced HO-1 promoter activity. Thus, PI3K is necessary to initiate activation of the HO-1 promoter through the AREs in an Nrf2-dependent manner [107].

However, Nrf2-independent HO-1 expression has also been reported [108]. Bach1 is regarded as a critical physiological repressor of HO-1. Higher levels of HO-1 mRNA were observed in the thymus, heart, lung, and liver in the bach1−/− mice [108]. Since HO-1 is activated by Nrf2 [95,96,97,98], higher levels of HO-1 expression in the bach1−/− mice seem to be due to Nrf2 activation. Surprisingly, the enhanced HO-1 levels in the Bach1-deficient thymus were independent of Nrf2, since the expression of HO-1 was not affected by Nrf2 deficiency [108]. In addition, CO/HO-1 induce NQO1 expression via Nrf2 activation [85], suggesting a crosstalk between HO-1 and Nrf2.

## 5. Small Molecule Natural Products Activating Nrf2-HO-1 Signaling

**Figure 3:**
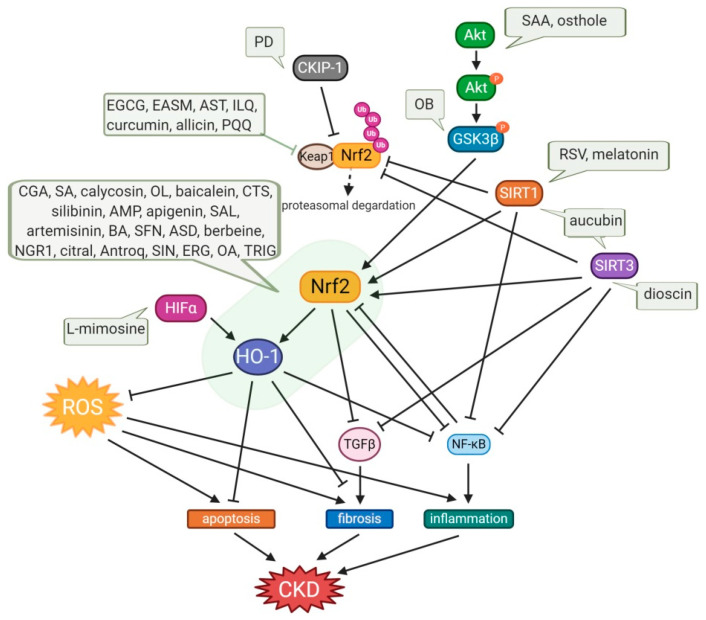
Protective effects of small-molecule natural products on OS in CKD. Osthole and SAA enhance the activation of the Akt/Nrf2/HO-1 signaling pathway with suppression of NF-kB and TGFβ1, consequently attenuating OS, inflammation, and fibrosis. OB induces the phosphorylation of GSK3β, which inhibits Fyn-mediated Nrf2 nuclear export, and activates the transcription of Nrf2-driven antioxidant genes. Expression of SIRT1, which inhibits NF-kB activity, and the activation of Nrf2 are enhanced by aucubin, melatonin, and RSV, which also upregulates SIRT3, resulting in amelioration of kidney injury. Dioscin upregulates SIRT3 level, promotes Nrf2, and suppresses Keap1 expression, resulting in inhibition of inflammation, lipid metabolism, OS, and kidney fibrosis. PD increases the CKIP-1 expression level and promotes the interaction of CKIP-1 with Nrf2, consequently activating the Nrf2-ARE antioxidative pathway. Allicin, AST, curcumin, EASM, EGCG, ILQ, and PQQ attenuate OS via the Nrf2/HO-1 signaling pathway with inhibition of Keap1, and they also reduce TGFβ-mediated fibrosis and NF-kB-induced inflammation. In the cases of an anti-fibrotic effect of apigenin, ASD, baicalein, BA, CGA, CTS, ERG, OL, and SFN, AMP, antroq, artemisinin, berbeine, calycosin, SA, SIN, and TRIG, they are mediated not only by upregulation of the Nrf2/HO-1 antioxidant signaling pathway and downregulation of NF-kB-induced inflammation, but also via TGFβ suppression. Treatments with citral, NGR1, OA, SAL, and silibinin have potency for anti-apoptotic effects with regulation of Bcl2/Bax and caspase3. The decrease in the NLRP3 inflammasome was also observed in treatments with baicalein, EGCG, and OL. L-mimosine activates HIF1α, which upregulates renoprotective HIF target genes, such as VEGF, HO-1, and GLUT1, and decreases fibrosis markers. AMP, ampelopsin; Antroq, antroquinonol; ASD, akebia saponin D; AST, astaxanthin; BA, betulinic acid; CGA, chlorogenic acid; CTS, cryptotanshinone; EASM, ethyl acetate extract of Salvia miltiorrhiza; EGCG, Epigallocatechin gallate; ERG, ergone; GSK3β, glycogen synthase kinase 3β; HIFα, hypoxia-inducible factor α; ILQ, isoliquiritin; NGR1, notoginsenoside R1; OA, oleanolic acid; OB, obacunone; OL, oleuropein; PD, polydatin; PQQ, pyrroloquinoline quinone; RSV, resveratrol; SA, sinapic acid; SAA, salvianolic acid A; SAL, salidroside; SFN, sulforaphane; SIN, sinomenine; TRIG, trigonelline.

A substantial quantity of natural products has been reported to confer renoprotection and improve disease outcomes of the various types of CKD, primarily through activating the Nrf2/HO-1 antioxidant defense systems and attenuating the proinflammatory signaling pathways. Here, we reviewed the existing literature over the past decade to compile comprehensive information on the kidney protective potential of naturally occurring compounds. Experimental and disease models, the pathobiology involved, the research outcomes, and the molecular markers altered by these compounds are summarized in Table 1 and Table 2 and Figure 3. To facilitate the discussion, we have categorized the kidney protective effects of these natural compounds into two distinct chemical groups: phenolic and non-phenolics. This categorization also highlights common bioactive compounds, belonging to phenolic group which represents the largest chemical class showing enormous bioactivity with the potential to be future drug candidates.

### 5.1. Phenolic Compounds

A significant number of phenolic compounds have shown protection against DKD (Table 1). Administration of resveratrol, a versatile bioactive phenolic found in many plant sources, including red grapes, peanuts, and berries, ameliorated diabetes-induced changes in the kidney tissues of STZ-induced rats by attenuating inflammatory signaling pathways through a mechanism that involved regulation of the NF-κB and Nrf2 signaling pathways [125]. Alone or in combination with metformin, salvianolic acid A attenuated diabetes-induced macrovascular and kidney injury in STZ-injected mice by activating the Nrf2/ARE pathways [129]. In a similar experimental setup, Gong and colleagues reported nephroprotective effects of a stilbenoid glucoside polydatin, which relieved HG-induced kidney damage through activating the CKIP-1-Nrf2-ARE pathway [153]. In STZ-injected mice, epigallocatechin gallate (EGCG) prevented diabetes-induced kidney damage by upregulating Nrf2 expression, which was mediated by disrupting the Nrf2-Keap1 complex [120]. Chlorogenic acid attenuated diabetes-induced kidney damage in STZ-injected and HFD-fed Sprague-Dawley rats by mitigating OS and inflammation through a mechanism that involved modulation of the Nrf2/HO-1 and NF-ĸB signaling pathways [115]. Astaxanthin attenuated HG-induced OS and FN accumulation in glomerular mesangial cells and improved the metabolic status and kidney morphology in STZ-induced diabetic rats [111]. These renoprotective activities of AST were attributed to its activation of Nrf2/ARE signaling [111]. Sinapic acid prevented STZ-induced DKD in rats by attenuating inflammation and OS through upregulating Nrf2/HO-1 signaling pathways [132]. Calycosin ameliorated kidney injury and dysfunction in HFD-fed/STZ-induced diabetic rats by inhibiting inflammation, and OS through modulating the IL33/ST2, NF-κB, and Nrf2 signaling pathways [114].

Wang et al. demonstrated restoration of kidney function by EGCG relieving oxidative and inflammatory damage in UUO, which is attributed to the regulatory roles of this common tea polyphenol on the NF-κB and Nrf2/HO-1 signaling pathways [121]. In a subsequent study, Wang et al. reported that the administration of cryptotanshinone at 50 and 100 mg/kg/day prevented OS and inflammation by suppressing NF-κB signaling and activating Nrf2 signaling in a mouse model of UUO [116]. Rotenone, a mitochondrial complex I inhibitor, ameliorated chronic obstructive kidney injury through attenuating mitochondrial OS, inflammation, and fibrosis [127].

Silibinin (75 mg/kg day) significantly reversed arsenic (As)-induced biochemical changes in the kidney, reduced lipid peroxidation, and improved the antioxidant defense system [131]. These nephroprotective effects of silibinin against As-induced CKD were attributed to its antioxidant, anti-inflammatory, and metal chelating properties [131]. Co-administration of osthole (40 mg/kg, intravenously) along with 2% adenine suspension attenuated inflammatory damage in a rat model of CKD through a mechanism that involved downregulation of NF-κB and TGFβ1 and activation of PI3K/Akt/Nrf2 signaling [124].

EGCG protected against LN in mice by activating the Nrf2 antioxidant signaling pathway and inhibiting the NLRP3 inflammasome [56]. In a similar study, Li et al. showed that baicalein improved pristane-induced LN symptoms in mice through preventing inflammation and OS by a mechanism that involved activation of the Nrf2/HO-1 signaling pathway and upregulation of NLRP3 expression [113]. Mice fed oleuropein- and peracetylated oleuropein-supplemented diets experienced a lower intensity of pristane-induced kidney damage. These protective effects of oleuropein and peracetylated oleuropein against LN were attributed to its activating role of HO-1/Nrf2 signaling and its suppressive effect on the JAK/STAT, NF-κB, MAPK, and NLRP3 inflammasome signaling pathways [123].

Curcumin protected against the changes in kidney functions in 5/6 nephrectomy, an experimental CKD model, through activating the Nrf2-Keap1 and kidney dopamine pathways, an effect that was comparable to the standard agent mycophenolate mofetil [117]. Curcumin ameliorated adenine-induced alteration of kidney functions, including hypertension and albuminuria, in a rat model of CKD by attenuating inflammation and OS through activating Nrf2 signaling [118]. Liu et al. demonstrated kidney protective effects of isoliquiritin against cationic BSA-induced MGN in an experimental rat model, which was attributed to its antioxidative (activation of Nrf2 signaling) and anti-inflammatory properties (inhibition of NF-κB signaling) [54].

Moreover, resveratrol, curcumin, ampelopsin, and apigenin were shown to have attenuating effects against oxidative damage in various cellular models of kidney injury [109,110,119,126]. Besides, salidroside has anti-apoptotic effects in HG-treated mouse podocytes, showing therapeutic promise in the management of kidney disease [128].

### 5.2. Non-Phenolic Compounds

Like phenolics, several non-phenolic compounds have been shown to protect against DKD (Table 1). Most notable is sulforaphane, which improved kidney morphological and functional alterations in STZ-injected and meglumine diatrizoate-injected diabetic rats through activation of the Nrf2/HO-1 pathway [150]. Sulforaphane treatment also resulted in functional and morphological improvements of CRAD by attenuating OS through inducing the Nrf2-HO-1/Nqo1 signaling pathway [151]. Artemisinin prevented OS-induced kidney damage in STZ-injected DN. These renoprotective effects of artemisinin were due to its inhibitory role on the TGFβ1 regulator and its activating role in the Nrf2 signaling pathway [136]. Betulinic acid ameliorated DKD in STZ-induced rats, which was mediated by activating the AMPK/NF-κB/Nrf2 signaling pathway [139]. Akebia Saponin D protected against diabetes-induced kidney damage and improved kidney function by antioxidant and anti-inflammatory functions, which was attributed to its activation of the Nrf2/HO-1 pathway and its inhibition of the NF-κB pathway [133]. In STZ-induced diabetic mice, berberine can ameliorate tubulointerstitial fibrosis via activating the Nrf2 pathway and inhibiting the TGFβ/Smad/EMT signaling pathway [138]. Aucubin, a natural iridoid glucoside, improved symptoms of DKD through inhibiting NF-κB activation and inducing the SIRT1/SIRT3-FOXO3a signaling pathway in HFD/STZ-induced diabetic mice [137]. Notoginsenoside R1 protected against OS and ameliorated DKD in db/db mice through upregulation of Nrf2-mediated HO-1 expression [145].

Sinomenine ameliorated kidney fibrosis in UUO-operated ICR mice by preventing OS through Nrf2 activation and interfering with pro-fibrogenic signaling of TGFβ/Smad and Wnt/β-catenin [149]. Allicin can protect against 5/6 nephrectomy-induced hypertension and kidney dysfunction (uremia, high serum creatinine, and albuminuria) through activating the Nrf2/Keap1 antioxidant defense system, an effect that was similar to, or even better, than that of losartan [134]. Administration of L-mimosine at a later stage of kidney ablation (from week 5 to week 12) caused transient activation of hypoxia-inducible factors (HIF-1α and HIF-2α proteins), increased expression of VEGF, HO-1, and GLUT-1, and a reduction in fibrosis markers [143].

Dioscin prevented high fructose-induced kidney damage via attenuating SIRT3-mediated OS and inflammation and adjusting lipid metabolism and TGFβ1/Smad signaling to inhibit kidney fibrosis [141]. When treated with ergone, alisol B 23-acetate, and pachymic acid B, these compounds prevented ECM accumulation in HK-2 cells and attenuated podocyte injury by inhibiting Ang II-induced RAS/Wnt/β-catenin axis activation and thereby ameliorating tubulointerstitial nephropathy [142]. Oleanolic acid treatment of cyclosporine-treated ICR mice ameliorated tubulointerstitial fibrosis in chronic nephropathy by activating the Nrf2/HO-1 signaling pathway [147]. Trigonelline can prevent the effects of oxalate-induced EMT in kidney tubular epithelial cells, offering a promising anti-fibrotic agent in the management of CKD [152].

Melatonin can protect against pristane-induced LN in mice, and this effect was attributed to its enhancing role on the Nrf2 signaling pathway and its ability to inhibit kidney NLRP3 inflammasome activation [144]. Also, obacunone and pyrroloquinoline quinone have shown promise in CKD for their antioxidant and anti-inflammatory potential [146,148].

## 6. Conclusions and Future Perspectives

OS has been involved in the pathobiology of CKD and thus, developing a treatment strategy targeting OS might be a potential option against CKD. Cells have their own antioxidant defense system to tackle the effects of OS, and Nrf2-HO-1 signaling is an important antioxidant defense system against various diseases, including CKD. Several recent clinical trials are investigating the protective potential of phytochemicals, such as resveratrol, curcumin, and sulforaphane, in CKD patients. In those studies, the expression of Nrf2 and HO-1 were set as outcome measures (Table 3), although Nrf2 activates HO-1 under OS in many preclinical settings of CKD. Targeting Nrf2-HO-1 may provide a means of controlling OS. Pharmacological modulators that can activate Nrf2-HO-1 antioxidant systems offer promise for the treatment of diseases associated with OS-associated kidney injury. In this perspective, several phytochemicals have been described to protect against kidney injury by activating Nrf2-HO-1 systems, suggesting that they could be used to design novel therapeutic agents for treating CKD.

Although the kidney protective actions of the mentioned phytochemicals are promising, their protective effects have only been studied in preclinical settings. Although few clinical trials are ongoing, they may fail in clinical studies. For instance, resveratrol has been proven to be a potent kidney protective agent that activates the Nrf2-HO-1 pathway in various cells, but it has shown poor bioavailability. Thus, it needs advanced drug delivery systems, such as nanoparticle-mediated drug delivery, in order to achieve proper doses of the drug. Likewise, investigating the detailed molecular mechanism of the kidney protective effects of these phytochemicals is important to discover which cellular defense system between the Nrf2 and HO-1 pathways is involved. Also, it would be useful to study the pharmacokinetics as well as pharmacodynamics of these phytochemicals on the gender differences in CKD.

## Figures and Tables

**Table 1:** Kidney protective effects provided by phenolic compounds of phytochemicals targeting the Nrf2-HO-1 signaling pathway.

No.	Modulator	Chemical Class and Natural Sources	Experimental Model	Disease Model	Pathobiology Involved	Major Research Outcomes	Molecular Markers	Ref.
Phenolic compounds								
1	Ampelopsin	Flavonoid; *Ampelopsis grossedentata*	HG-stimulated hGMCs	OS	OS, ECM accumulation	Amelioration of OS and ECM accumulation	↓ROS, ↓MDA, ↑SOD, ↓Nox2, ↓Nox4, ↓NADPH, ↓FN, ↓Col IV, ↑n-Nrf2, ↑HO-1,	[109]
2	Apigenin	Flavonoid; common fruits and vegetables	HG-treated HK-2 cells	Oxidative damage	Oxidative damage	Decrease in apoptosis, inhibition of OS, and inflammatory response	↓LDH, ↓MDA, ↑SOD, ↑CAT, ↓TNFα, ↓IL-1β, ↓IL-6, ↑Nrf2, ↑HO-1	[110]
3	Astaxanthin	Xanthophyll carotenoid; algae, shrimp, lobster, crab, salmon, and other organisms	STZ-injected rat	DKD	ECM accumulation	Amelioration of kidney injury	↓FN, ↓TGFβ1, ↓ICAM-1	[111]
			HG-treated GMCs	Kidney fibrosis	OS	Increase in antioxidative capacity	↓FN, ↓TGFβ1, ↓ICAM-1, ↑SOD, ↓MDA, ↓ROS, ↓DHE, ↑n-Nrf2, ↓keap1, ↓SOD-1, ↓Nqo1, ↓HO-1	
			Adriamycin-treated BALB/c mice	FSGS	OS, inflammation	Anti-inflammation, antioxidation	↓TGFβ1, ↓collagen1, ↓α-SMA, ↓MDA, ↑GSH, ↑SOD, ↑CAT, (serum: ↓IL-1 β, IL-18), ↑Nrf2, ↓NLRP3	[112]
4	Baicalein	Flavonoid; roots of *Scutellaria baicalensis Georgi*	Pristine -injected BALB/c mice	LN	OS, inflammation	Attenuation of kidney dysfunction, antioxidation, anti-inflammation, inhibition of MDSC expansion	↓IL-1b, ↓IL-18, ↓O_2_^¯^˙, ↑ GPx, ↑Nrf2, ↑HO-1, ↓ NLRP3, ↓Casp-1, ↓mIL-1 β, ↓p-NF-kB	[113]
			LPS-primed spleen-derived MDSCs	OS, inflammation			↓ROS, ↓IL-1β, ↓IL-18, ↑Nrf2, ↑HO-1, ↓NLRP3, ↓mIL-1β/pro-IL-1β, ↓Casp-1-p20/pro-casp-1-p45, ↓p-NF-kB/NF-kB, ↓Ang-1, ↓p47phox, ↓GP91phox, ↓iNOS	
5	Calycosin	Isoflavone; root of *Astragalus membranaceus*	HFD-fed/ STZ-injected SD rat	DKD	Inflammation, OS, fibrosis	Inhibition of inflammatory, oxidative, and fibrotic events	↓IL-33, ↓ST2, ↓NF-kB p65, ↓TNFα, ↓IL-1 β, ↓IL-6, ↑Nrf2, ↓MDA, ↓TGFβ	[114]
6	Chlorogenic acid	Cinnamate ester; coffee, fruits, and vegetables	STZ-injected and HFD-fed SD rat	DKD	OS, inflammation	Relieve kidney injury, mitigation of OS, inflammation	↓MDA, ↑SOD, ↑GSH-Px, ↑n-Nrf2, ↑HO-1, ↓IL-6, ↓TNFα, ↓IL-1 β, ↑c-NF-kB, ↓n-NF-kB, ↑IkBα, ↓p-IkBα,	[115]
			HG-treated rat mesangial cell line (HBZY-1)			Mitigation of OS, inflammation, increase in cell proliferation	↑n-Nrf2, ↑HO-1, ↑c-NF-kB, ↓n-NF-kB, ↑IkBα, ↓p-IkBα, ↓IL-6, ↓TNFα, ↓IL-1 β	
7	Cryptotanshinone	Quinoid diterpene; *Salvia miotiorrhiza* bunge	UUO-operated mice	Kidney fibrosis	OS, inflammation	Attenuation of OS and inflammation	↓collagen-1, ↓FN, ↓CD68, ↓CD3, ↑IkBα, ↓NF-kB p65, ↑SOD2, ↑CAT, ↑GSH, ↓MDA, ↑Nuclear Nrf2, ↓cytosolic Nrf2, ↑HO-1	[116]
8	Curcumin	Curcuminoid; turmeric (*Curcuma longa*)	5/6 nephrectomy Wistar rat	CKD	OS, inflammation	Protection of kidney function, antioxidant, anti-inflammation	↓Nox4, ↑eNOS, ↓nitrotyrosine, ↓MCP-1, ↓Keap-1, ↑Nrf2, ↑GPx-1, ↑CAT, ↑SOD-1, ↓phospho serine D1R	[117]
			0.25% Adenine -diet rat	CKD	OS, inflammation	Amelioration of kidney function and OS	↓IL-1 β, ↓IL-6, ↓TNFα, ↑cycstatin C, ↓adiponecitn, ↑sclerostin, ↑SOD, ↑Nrf2, ↑GSH reductase. ↓ caspase3	[118]
			HG-treated NRK-52E cells	OS	OS	Increase in cell viability, inhibition of EMT	↑E-cadherin, ↓α-SMA, ↑Nrf2, ↑HO-1	[119]
9	Epigallocatechin-3 -Gallate	Polyphenol; Dried leaves of tea plant (*Camellia sinensis*)	STZ-injected mice	DKD	Oxidative damage, inflammation,	Anti-OS	↓TGFβ1, ↓PAI-1, ↓ICAM-1, ↓VCAM-1, ↓MDA, ↓iNOS, ↓3-NT, ↑Nqo1, ↑HO-1, ↑t-Nrf2, ↑c-Nrf2, ↑n-Nrf2, ↑n-Nrf2/t-Nrf2	[120]
			HG-cultured MMC				↑t-Nrf2, ↑c-Nrf2, ↑n-Nrf2, ↑Nqo1, ↑HO-1, ↓MDA, ↓iNOS, ↓VCAM-1, ↓ICAM-1, ↓COL4, ↓FN	
			NZB/W F1 lupus-prone mice	LN	OS	Antioxidant and anti-inflammation	↑Nrf2, ↓p47phox, ↑Nqo1, ↑HO-1, ↑GPx, ↓CD3, ↓F4/80, ↓NF-kB, ↓NLRP3, ↓IL-1 β, ↓IL-18, ↓casp1-p20,	[56]
			UUO mice	CKD	OS, inflammation	Kidney function improvement, prevention of OS and inflammation	↑SOD, ↑CAT, ↑GSH-Px, ↓MPO, ↓TNFα, ↓IL-6, ↓IL-1 β, ↑IkBα, ↓p-IkBα, ↓NF-kB, ↑n-Nrf2, ↑HO-1, ↑t-bilirubin	[121]
10	Ethyl acetate extract of *Saliva miltiorrhiza*	Diterpenoids, phenolic compounds, flavonoids, triterpenoids; dried root of *Salvia miltiorrhiza* Bunge	STZ-injected mice	DKD	Oxidative stress	Antioxidation, attenuation of kidney dysfunction	↑Nrf2, ↑HO-1, ↑Nqo1, ↓Keap1	[122]
			HG-treated SV40-MES-13 MMCs	hyperglycemia		Antioxidation	↓ROS, ↑Nrf2, ↑HO-1, ↑Nqo1, ↓Keap1	
11	Isoliquiritin	Flavonoid glycoside; Chinese licorice (*Glycyrrhiza uralensis*)	Cationic BSA-injected SD rat	MGN	Inflammation and OS	Antioxidative, anti-inflammatory activities	↓Keap1, ↑Nrf2, ↓n-Nrf2, ↑c-Nrf2, ↑HO-1, ↑Nqo1, ↓MDA, ↓NO, ↑SOD, ↑CAT, ↑GPx, ↑GSH, ↓NF-kB p65, ↓nuclear NF-kB p65, ↑cyclic NF-kB, ↓IKKb, ↓p-IKKb, ↓TNFα, ↓IL-1 β, ↓COX2, ↓iNOS, ↓p38 MAPK, ↓p-p38 MAPK	[54]
12	Oleuropein, peracetylatedoleuropein	Secoiridoid; olive leaves, roots, and unprocessed olive drupes	Pristine -injected BALB/c mice	LN	Inflammation and OS	Amelioration of kidney abnormalities, inhibition of proinflammation, antioxidation	↓MMP-3, ↓iNOS, ↓mPGEs-1, ↓PGE2, ↑Nrf2, ↑HO-1, ↓pSTAT3, ↓NF-kB-p65, ↑IkBα, ↓pp38, ↓pJNK, ↓pERK1/2 ↓NLRP3, ↓ASC, ↓IL-18, ↓ IL-1β, ↓cleaved caspase-1, ↓cleaved caspase 11	[123]
13	Osthole	Coumarin; *Fructus Cnidii*	2% adenine suspension -received rat	CKD	Inflammation	Protection of kidney function, antiinflammation	↓TNFα, ↓IL-6, ↓IL-8, ↓NF-kB/p65, ↓TGFβ1, ↓MCP-1, ↑p-Akt/Akt, ↑Nrf2	[124]
14	Polydatin	Stilbenoid glucoside; *Polygonum cuspidatum* Sieb.et Zucc	STZ-injected diabetic mice	DKD	OS	Improvement of antioxidative effect and kidney dysfunction	↑CKIP-1, ↑Nrf2, ↑HO-1, ↑SOD1, ↓FN, ↓ICAM-1, ↓MDA, ↑t-SOD	
			HG-treated rat GMCs				↑Nrf2, ↓Keap1, ↑n-Nrf2, ↓n-CKIP-1, ↑ARE binding activity, ↑HO-1, ↑SOD1, ↓DHE, ↓H_2_O_2_, ↓FN, ↓ICAM-1	
15	Resveratrol	Phytoalexin; red grapes (*Vitis vinifera* L.), peanuts (*Arachis* spp.), berries (*Vaccinium* spp.)	STZ-induced Wistar rat	DKD	OS	Anti-inflammation, Anti-OS	↓iNOS, ↓NF-kB, ↓Nrf2, ↓NGAL, ↓IL-1β, ↓IL-6, ↓IL-8, ↓TNFα	[125]
			4-hydroxy-2-hexenal-treated mouse cortical collecting duct cells (M1)	OS			↓nuclear p65, ↑cytosol IkB, ↑SIRT1, ↓Nox4, ↓COX2, ↑AQP2, ↓pERK/ERK, ↓pJNK/JNK, ↓pP38/P38, ↓Nrf2, ↑Keap1	[126]
16	Rotenone	Isoflavonone; seeds and stems of jicama vine plant, the roots of Fabaceae, etc.	UUO-operated mice	Kidney fibrosis	Mitochondrial abnormality	Anti-OS, anti-inflammation, anti-fibrosis	↓TBARS, ↓HO-1, ↓TNFα, ↓IL-1β, ↓ICAM1, ↓collagen I, ↓FN, ↓α-SMA, ↓PAI-1, ↓collagen III, ↓TGFβ, ↑mtDNA, ↑mtNd1	[127]
17	Salidroside	phenylpropanoid glycoside; plant *Rhodiola rosea*	HG-treated mouse podocytes	Apoptosis	Apoptosis	Improvement of cell viability	↓Caspase-9, ↓caspase-3, ↑HO-1, ↑p-ILK/ILK, ↑p-Akt/Akt, ↑p-ERK/ERK, ↑p-JNK/JNK, ↓p-p38/p38, ↑Nrf2	[128]
18	Salvianolic acid A	Polyphenol derivative; root of *Salvia miltiorrhiza*	STZ-injected mice	DKD	OS	Anti-OS	↓VCAM-1, ↑HO-1, ↓α-SMA, ↓NT, ↓DHE, ↑GPx-1	[129]
			HG-treated HK-2 cells				↑HO-1, ↓α-SMA, ↓p65, ↓ROS	
			5/6 nephrectomized SD rats	CKD	OS	OS attenuation,	↑t-SOD, ↑GPx, ↑CAT, ↓MDA, ↓ROS, ↓Nox4, ↑p-Akt/Akt, ↑p-GSK3β/GSK3β, ↑p-Nrf2/Nrf2, ↑HO-1	[130]
			H_2_O_2_-treated/LPS-treated HK-2 cells			Cell viability improvement, decrease in OS	↑t-SOD, ↑GPx, ↑CAT, ↓MDA, ↓ROS, ↓Nox4, ↑p-Akt/Akt, ↑p-GSK3β/GSK3β, ↑n-Nrf2, ↑HO-1, ↓p-NF-kB p65/NF-kB p65, ↓ICAM-1, ↓p-NF-kB p65, ↓ICAM-1, ↑n-Nrf2, ↑HO-1	
19	Silibinin	Flavonoliganas: milk thistle seeds	Arsenic -induced rat	CKD	Inflammation	Attenuation of OS, inflammation, and apoptosis	↓TNFα, ↓iNOS, ↓NO, ↓NF-kB, ↓Caspase-3, ↓NADPH oxidase, ↑Nrf2	[131]
20	Sinapnic acid	Hydroxycinnamic acid; wine, vinegar	STZ-injected rat	DKD	OS, inflammation	Amelioration of OS and inflammation	↑CAT, ↑GPx, ↑SOD, ↓TNFα, ↓IL-6, ↓NO_2_, ↓MDA, ↓TFGβ, ↑HO-1, ↑Nrf2, ↓NF-kB, ↑IkBα, ↑Bcl2, ↓Caspase3, ↓Bax	[132]

AQP2, aquaporin 2; α-SMA, α-smooth muscle actin; BSA, bovine serum albumin; CAT, catalase; CKD, chronic kidney disease; COX2, cyclooxygenase; DHE, dihydroethidium; DKD, diabetic kidney disease; ECM, extracellular matrix; EMT, epithelial-to-mesenchymal transition; eNOS, endothelial nitric oxide synthase; FN, fibronectin; GMCs, glomerular mesangial cells; GPx, glutathione peroxidase; GSK3β, glycogen synthase kinase 3β; HFD, high fat diet; HG, high glucose; HO-1, Heme oxygenase-1; ICAM, intercellular adhesion molecule 1; iNOS, inducible nitric oxide synthase; LDH, lactate dehydrogenase; LN, lupus nephritis; LPS, lipopolysaccharide; MCP-1, monocyte chemoattractant protein-1; MDA, malondialdehyde; MDSCs, myeloid-derived suppressor cells; MGN, membranous glomerulonephritis; MMCs, mouse mesangial cells; NGAL, neutrophil gelatinase-associated lipocalin; NLRP3, NLR family pyrin domain containing 3; Nqo1, NADPH quinone oxidoreductase; Nrf2, nuclear factor erythroid 2-related factor 2; MPO, myeloperoxidase; NT, nitrotyrosine; OS, oxidative stress; PAI-1, plasminogen activator inhibitor-1; SOD, superoxide dismutase; STZ, streptozotocin; TBARS, thiobarbituric acid reactive substances; UUO, unilateral ureteral obstruction; VCAM, vascular cell adhesion molecule 1.

**Table 2:** Kidney protective effects provided by non-phenolic compounds of phytochemicals targeting the Nrf2-HO-1 signaling pathway.

No.	Modulator	Chemical Class and Natural Sources	Experimental Model	Disease Model	Pathobiology Involved	Major Research Outcomes	Molecular Markers	Ref.
Non-phenolic compounds								
1	Akebia Saponin D	triterpenoid saponin; *Dipsaci Radix*	STZ-injected mice	DKD	OS, inflammation	Amelioration of kidney damage, inflammation, OS, and apoptosis	↓TNFα, ↓IL-1β, ↓IL-6, ↓MCP-1, ↓ROS, ↓MDA, ↓LDH, ↑SOD, ↑Bcl2, ↓Bax, ↓cleaved caspase3/caspase3, ↓cleaved caspase9/caspase9, ↑n-Nrf2, ↓p-NF-kB/t-NF-kB, ↑HO-1, ↑Nqo1, ↓p-IkBα/t-IkBα	[133]
			HG-treated HK-2 cells				↓TNFα, ↓IL-1β, ↓IL-6, ↓MCP-1, ↓ROS, ↓MDA, ↓LDH, ↑SOD, ↑Bcl2, ↓Bax, ↓cleaved caspase3/caspase3, ↓cleaved caspase9/caspase9, ↑Nrf2, ↓p-NF-kB/t-NF-kB, ↑HO-1, ↑Nqo1, ↓p-IkBα/t-IkBα	
2	Allicin	Diallyl thiosulfinate; garlic (*Allium sativum* L.)	5/6 nephrectomy Wistar rat	CKD	Fibrosis, OS	Antihypertensive and antioxidant effects	↑AT1R, ↑AT2R, ↑Nrf2, ↓Keap1, ↑CAT, ↑SOD, ↓HO-1, ↑eNOS	[134]
3	Antroquinonol	Enone; mushroom (*Antrodia camphorate*)	Adriamycin -injected BALB/c mice	FSGS	OS	Decrease in kidney dysfunction, anti-OS, anti-inflammation	↓desmin, ↓O_2_^●−^, (serum, urine ↓ O_2_^●−^, ↓NO), ↓DHE, ↓p47phox, ↑Nrf2, ↑GPx, ↓NF-kB p65, ↓MCP-1, ↓IL-6, ↓CD3, ↓F4/80, ↓Col I, ↓Col III, ↓Col IV, ↓TGFβ1	[135]
4	Artemisinin	sesquiterpene lactones; *Asteraceae Artemisia annua*	STZ-injected rat	DKD	OS	Amelioration of kidney dysfunction and OS	↓MDA, ↑t-SOD, ↑GPx, ↓TGFβ1, ↑t-Nrf2, ↑n-Nrf2, ↑HO-1, ↑Nqo1	[136]
5	Aucubin	iridoid glycoside; leaf of *Eucommia ulmoides*	HFD-fed and STZ-injected mice	DKD	OS, inflammation	Amelioration of kidney dysfunction, anti-inflammation, anti-OS	↓FN, ↓collagen IV, ↓MDA, ↑SOD, ↑CAT, ↑GSH/T-GSH, ↓TNFα, ↓IL-6, ↓IL-1β, ↓p65, ↓IkBα, ↑Nrf2, ↑HO-1, ↑Nqo1, ↑FOXO3α, ↓p-FOXO3α/FOXO3α, ↑SIRT1, ↑SIRT3, ↓Ac-FOXO3α/FOXO3α	[137]
6	Berberine	isoquinoline alkaloid; *Coptidis Rhizoma* and *Cortex Phellodendri*	STZ-injected mice	DKD	OS	Anti-fibrosis	↓α-SMA, ↓collagen-1, ↑Nrf2, ↑NQO1, ↑HO-1	[138]
			HG-treated NRK 52E cells	EMT			↓E-cadherin, ↓α-SMA, ↑n-Nrf2, ↑Nqo1, ↑HO-1, ↓p-Smad2, ↓p-Smad3	
7	Betulinic acid	pentacyclic triterpenoid; from the outer bark of white birch trees (*Betula alba*)	STZ-injected SD rat	DKD	OS	Anti-OS	↓IL-1 β, ↓IL-6, ↓MDA, ↑SOD, ↑CAT, ↑p-AMPK/AMPK, ↓p-IkBα/IkBα, ↓p-NF-kB/NF-kB, ↑Nrf2, ↑HO-1	[139]
8	Citral	Terpeonids; *Litsea cubeba*	Adriamycin -injected BALB/c mice	FSGS	OS	Amelioration of kidney dysfunction, anti-OS, anti-inflammation, anti-apoptosis	↓O_2_^¯^˙, (serum, urine ↓O_2_^¯^˙, ↓NO), ↓DHE, ↓p47phox, ↑Nrf2, ↑Nqo1, ↑HO-1, ↓desmin, ↓TUNEL, ↓Casp-3p17, ↓Casp-9p37, ↓Bax/Bcl2, ↓pNF-kB p65, ↓MCP-1, ↓ CD3, ↓F4/80	[140]
			LPS-treated RAW 264.7 macrophages	OS			↓NO, ↓NF-kB, ↓IL-6, ↓TNFα, ↓IL-1β, ↓p-ERK1/2(10min), ↓p-JNK1/2(15,30min)	
9	Dioscin	Steroid saponin; *Dioscoreae rhizoma*	10% fructose -fed mice	CKD	Oxidative damage, lipid metabolism, fibrosis	Inhibition of inflammation, lipid metabolism, OS, kidney fibrosis	↓MDA, ↑SOD, ↑GSH-Px, ↓α-SMA, ↑SIRT3, ↑SOD2, ↓IL-1β, ↓IL6, ↓TNFα, ↓NF-kB, ↓HMGB1, ↓COX2, ↓c-Jun, ↓c-Fos, ↓SREBP-1c, ↓SCD-1, ↓FASn, ↓p-Akt, ↓p-FoxO1A, ↓ACC, ↑CPT1, ↑Nrf2, ↓Keap1, ↑GST, ↓TGFβ1, ↓p-Smad3, ↑Smad7	[141]
10	Ergone (alisol B 23-acetate, pachymic acid B)	steroid; *Polyporus umbellatus*, surface layer of *Poria cocos, Alisma orientale*	AngII- treated HK-2 and conditionally immortalized MPC5 cells	CKD	OS, inflammation, impaired Nrf 2 activation	inhibition of the RAS/Wnt/b-catenin signaling cascade	(HK-2) ↓Snail1, ↓MMP-7, ↓Twist, ↓FSP-1, ↓Col I, ↓Col III, ↓α-SMA, ↓vimentin, ↑E-cadherin, ↓NF-kB, ↓MCP-1, ↓COX2, ↑Nrf2, ↑HO-1 (podocyte) ↓Snail1, ↓MMP-7, ↓Twist, ↓FSP-1, ↑podocin, ↑nephrin, ↑podocalyxin, ↑synaptopodin, ↓desmin, ↑WT1, ↓Akt2, ↓NF-kB, ↓MCP-1, ↓COX2, ↑Nrf2, ↑HO-1	[142]
11	L-mimosine	Amino acid; *Mimosa pudica*	Rats with remnant kidneys after subtotal nephrectomy (5/6 nephrectomy)	CKD	Fibrosis	Improvement of kidney function, inhibition of fibrosis	↑HIF-1α, ↑HIF-2α, ↑VEGF, ↑HO-1, ↑GLUT-1, ↓α-SMA, ↓collagen III	[143]
12	Melatonin	Endogenous indoleamine, coffee, walnut, etc.	Pristine -injected BALB/c mice	LN	OS, inflammation	Attenuation of OS, inflammation	↑SIRT1, ↑Nrf2, ↓TNFα, ↓NF-kB, ↓iNOS, ↓NLRP3, ↑CD31	[144]
13	Notoginsenoside R1	Saponin; *Panax notoginseng*	db/db mice	DKD	OS	Anti-OS, decrease in apoptosis	↓Collagen I, ↓TGFβ1, ↑Nrf2, ↑HO-1, ↓Bax/Bcl2, ↓Caspase-3, ↓Caspase-9	[145]
			AGEs-treated HK-2 cells	Mitochondria injury			↓LDH, ↓ROS, ↑n-Nrf2, ↑HO-1, ↓Bax/Bcl2, ↓Cspase-3, ↓Caspase-9, ↓TGFβ1, ↓collagen I	
14	Obacunone	Triterpenoid limonoid; citrus and other plants of the *Rutaceae* family	HG-treated NRK-52E cells	OS	OS	Inhibition of OS, mitochondrial injury, and apoptosis	↑SOD, ↑GSH, ↑CAT, ↓ROS, ↓JC-1 monomer/aggregate, ↑p-GSK3β/GSK3β, ↓n-Fyn, ↑n-Nrf2, ↑Nqo1, ↑HO-1, ↑SOD, ↑GSH, ↑CAT, ↓c-CytC/m-CytC, ↓cleaved caspase3	[146]
15	Oleanolic acid	Triterpenoid; olive oil, *Phytolacca Americana, Syzygium* spp, garlic, etc.	Cyclosporine -treated ICR mice	Chronic nephropathy	Inflammation, fibrosis	Antioxidation, anti-inflammation	↓α-SMA, ↑HO-1, ↑nuclear/total Nrf2, ↑SOD1, ↓MDA, ↓urinary 8-iso-PGF2α, ↓urine 8-oxo-dG, ↓Bax/Bcl2, ↓active caspase-3	[147]
16	Pyrroloquinoline quinone	In soil and foods such as kiwifruit and human breast milk	HG-treated HK-2 cells	OS	OS	Decrease in OS, inflammation and cellular senescence	↓IL-1β, ↓TNFα, ↓NF-kB, ↓p16, ↓p21, ↓ROS, ↑SOD2, ↑CAT, ↓keap1, ↑Nrf2, ↑HO-1, ↑Nqo1, ↑GST, ↑GPx3,	[148]
17	Sinomenine	Alkaloid; *Sinomenium acutum*	UUO-operated ICR mice	CKD	Fibrosis, OS	Anti-fibrosis, antioxidation	↑E-cadherin, ↓α-SMA, ↓FN, ↑HO-1, ↑Nqo1, ↑Nrf2, ↑SOD, ↑GPx, ↑CAT, ↑SOD2, ↓p-Smad3, ↓β-catenin	[149]
			TGFβ-treated/H_2_O_2_-treated HEK293 cells, TGFβ-treated RAW264.7 cells				↑E-cadherin, ↓α-SMA, ↓FN, ↑HO-1, ↑Nqo1, ↑Nrf2, ↑SOD, ↑GPx, ↑CAT, ↑SOD2, ↓p-Smad3, ↓β-catenin	
18	Sulforaphane	Isothiocyanate (organosulfur compound); Cruciferous vegetables such as broccoli, brussels sprouts, and cabbages	STZ-injected and meglumine diatrizoate-injected Wistar rats	DKD, CIN	OS	Renoprotective	↓MDA, ↓8-oxo-dG, ↑Nrf2, ↑HO-1, ↓IL6, ↑Caspase3	[150,151]
			Meglumine diatrizoate-treated NRK-52E cells			Cell viability	↑Nrf2, ↑HO-1, ↓IL6	
			F344 rat kidneys transplanted Lewis rat	CRAD	OS	OS alleviation, kidney functional and morphological improvements	↓MDA, ↓8-isoprostane, ↓ox-LDL, ↓8-oxo-dG, ↑SOD, ↑CAT, ↑GPx, ↑GR, ↑ γ-GCS, ↑Nrf2, ↑HO-1, ↑Nqo-1	[151]
19	Trigonelline	Alkaloid; traditional herbs (especially fenugreek), coffee bean, soybean, and other edible food plants	Oxalate-induced MDCK cells	EMT	Fibrosis	Attenuation of EMT, prevention of cell migration and ROS overproduction,	↓FN, ↓vimentin, ↓α-SMA, ↑ E-cadherin, ↑ZO-1, ↓MMP9, ↓ROS, ↑Nrf2	[152]

AGEs, advanced glycation end products; AngII, angiotensin II; α-SMA, α-smooth muscle actin; AT1/2R, angiotensin II receptor type 1/2; CAT, catalase; CIN, contrast induced nephropathy; CKD, chronic kidney disease; COX2, cyclooxygenase 2; CRAD, chronic renal allograft dysfunction; DHE, dihydroethidium; DKD, diabetic kidney disease; EMT, epithelial-to-mesenchymal transition; eNOS, endothelial nitric oxide synthase; FSGS, focal segmental glomerulosclerosis; γ-GCS, γ-glutamine cysteine synthase; GPx, glutathione peroxidase; GR, glutathione reductase; GSK3β, glycogen synthase kinase 3β; GST, Glutathione-S-transferase; HFD, high fat diet; HG, high glucose; HIF, hypoxia-inducible factor; HMGB1, high-mobility group box 1; HO-1, Heme oxygenase-1; iNOS, inducible nitric oxide synthase; LDH, lactate dehydrogenase; LN, lupus nephritis; LPS, lipopolysaccharide; MCP-1, monocyte chemoattractant protein-1; MDA, malondialdehyde; MDCK, Madin-Darby canine kidney; MMP, matrix metalloproteinase; NLRP3, NLR family pyrin domain containing 3; Nqo1, NADPH quinone oxidoreductase; Nrf2, nuclear factor erythroid 2-related factor 2; OS, oxidative stress; ox-LDL, oxidized low-density lipoprotein; RAS, renin-angiotensin system; SOD, superoxide dismutase; STZ, Streptozotocin; UUO, unilateral ureteral obstruction.

**Table 3:** Antioxidant natural compounds used for treating CKD patients.

Compound		Conditions or Disease	Phase of Clinical Trials	Outcome Measures	Clinical trials.gov Identifier or Ref.
Curcumin	Curcumin (320 mg/day, for 8 weeks)	CKD	Phase III (2013.2-2014. 4, 101 participants)	Oxidative stress markers (MDA, GSH, GSSG), antioxidants (GPx, SOD, CAT, Nrf2 activity.	NCT01831193 [154]
	Curcumin supplementation (500 mg of curcumin and 5 mg of piperine/day, for 12 weeks)	CKD	Not applicable (2018.2–2021.12, 31 participants)	Antioxidants (Nrf2, GPx, HO-1) and inflammatory biomarkers (NF-kB, IL-6, TNFα) in blood samples	NCT03475017 [155]
	Curcumin supplementation (500 mg of curcumin and 5 mg of piperine/day, for 12 weeks)	CKD, Peritoneal dialysis, hemodialysis	Not applicable (2020.10–2021.10, 30 participants)	Antioxidants (Nrf2, GPx, HO-1) and inflammatory biomarkers (NF-kB, IL-6, TNFα, CRP, IL-18, TBARS, inflammasome) in blood samples	NCT04413266
	Curcumin, NFE2L2 A > G (400 mg/2 times/day, for up to 24 weeks)	CKD, Type 2 diabetes mellitus, Polymorphism	Phase II/III (2018.8–2019.4, 176 participants)	Antioxidants (Nrf2, SOD, HO-1, GPx)	NCT03262363
Resveratrol	Resveratrol (500 mg/day, for 4 weeks)	CKD	Phase III (2013.01–2014.12, 20 participants)	Antioxidants (Nrf2, GPx, HO-1) and inflammatory biomarkers (NF-kB, IL-6, TNFα) in blood samples	NCT02433925
	Resveratrol 200 mg/2 times/day, for 6 weeks)	CKD, Endothelial dysfunction	Not applicable (2019.1–2021. 8, 25 participants)	Oxidative stress	NCT03597568
Sulforaphane	Sulforaphane (4 g L-sulforaphane/day, for 2 months + 4 g corn starch colored with chlorophyll /day, for 2 months)	CKD	Not applicable (2021.1–2022.12, 122 participants)	Antioxidants (Nrf2, GPx, HO-1) and inflammatory biomarkers (NF-kB, IL-6, TNFα) in blood samples	NCT04608903

CAT, catalase; CKD, chronic kidney disease; CRP, C-reactive protein; GPx, glutathione peroxidase; GSH, reduced glutathione; GSSG, oxidized glutathione; HO-1, Heme oxygenase-1; MDA, malondialdehyde; Nrf2, nuclear factor erythroid 2-related factor 2; SOD, superoxide dismutase; TBARS, thiobarbituric acid reactive substances.
